# Doctors in Fiction

**Published:** 1887-07-23

**Authors:** 


### Doctors in Fiction.

*(Continued from p. 263, vol. ii.)*

Of a very different type is Thackeray's other medical man, Dr. Firmin, the pretentious, shallow, false-hearted, would-be fashionable doctor, whom Pendennis hated so heartily and instinctively, although he was the father of his chum, that good-natured, bungling Philip, who by mere force of his straightforward manliness made friends among those who had most cause to hate his father. It is wonderful how readers of "Philip" come to share Pen's "Dr. Fell" aversion to Firmin, even before they know the full extent of his villany, before they learn that "the Little Sister" is his deserted wife. This Little Sister, Caroline, is the first specimen of the modern nurse we meet with in literature;—a most noble little woman altogether, brave and ready and skilful in the work she has undertaken; hiding her own sorrows under a smiling face, and patiently letting a shadow rest on her good name, rather than injure the son of the man who deceived and deserted her, by claiming her rightful position as his wife. Yes, Dr. Firmin deserved all the misfortune and ruin that befell him, if only for his treatment of the Little Sister.

As Thackeray contributed the first portrait of the new sick-nurse, Dickens gave us the last sketch of the old. A very amusing personage is Sairey Gamp, as we meet her in the pages of "Martin Chuzzlewit," but a very terrible personage she must have been to encounter anywhere else. Surely she is exaggerated! Photographic accuracy was a less distinguished mark of Dickens's work than an attractive but too highly coloured vividness, and this sacrifice of fidelity to effectiveness shows itself in his medical pictures. Mr. Bob Sawyer and Mr. Ben Allen must always have been exceptional, not to say mythical creatures, both in their student days and after the former made his remarkable start in practice. Albert Smith's medical student is much more true to life. He has his eccentricities, it is true; his occasional bursts of noisy jollity; but he is not a mere rowdy; and he really works at his profession, although in a burst of pre-examination terror he does speak of the nervous system—whose intricacy and beauty he so eulogised when first he began to study anatomy—as "a confounded nuisance," involving "a beastly grind," and of no use to him after he has passed his second professional.

Dickens has one serious and beautiful study of a medical man—Allan Weston, the young surgeon in "Bleak House," who teaches the dying crossing-sweeper, Jo, the Lord's Prayer. The laboured pathos of the author of "Dombey and Son" does not often touch so deep and true a note as sounds through this scene; but even here the doctor does not seem naturally, inevitably medical. Any other good man might have played the part; the picture is not painted by one who had studied doctors and their ways, and could show in half-hidden touches how the man had been affected by the profession he had adopted.

Such a remark cannot be applied to Trollope. He always knew his characters thoroughly. You may not like the characters, may think them uninteresting and commonplace, but you cannot deny that they are drawn to the life. But it would be difficult to avoid liking Dr. Thorne. He is such a straightforward, honourable gentleman; doing good as he has opportunity, and making no boast about it; incapable of a discourtesy, and equally incapable of bating a jot of justice or self-respect at the bidding of any human being. And he is thoroughly medical, devoted to his profession, although he has heart and thought to spare for the young people around him, and their trials and vexations. No reader of the novel will easily forget the aspect of his study, with its grinning skull staring at whoever came in, and the thigh-bones which the doctor had a habit of playing with when talking or thinking over some knotty point. Neither is it possible to forget the effect these decorations produced upon Lady Augusta's nerves when she interviewed the doctor about the love-affair between his niece Mary and her son Frank; the courage and condescension she thought she displayed in talking at all among such surroundings; and the complete rout to which she was put by Dr. Thorne's manliness and honesty.

Somewhat similar qualities mark Dr. May, the father of Miss Yonge's many-linked "Daisy Chain." He is a little old-fashioned, it is true; rather inclined to think that modern students push research to an absurd and unpractical extent; and at first not wholly in sympathy with his ardent, energetic, but opinionated son Tom, who, in his determination to increase his scientific knowledge, is perhaps unnecessarily inconsiderate towards his father's prejudices, as young folks are apt to be. It will be remembered as one of Dr. May's peculiarities that he could not bring himself to speak of the nurses who came to help, when the epidemic was devastating the town, as Sister Frances and Sister Ellen. He compromised with the new custom by alluding to them always as "those blessed women."

The most elaborate study of a doctor extant is also the saddest—George Eliot's portrait of Lydgate in "Middlemarch." She shows us a man full of noble ambitions; thoughtful, studious, and devoted to his profession; whose whole career is spoiled by the shallow selfishness of a wife who understands nothing but the outward shows of life. Success means different things in the minds of husband and wife. To her it stands for money and social consideration, and an existence carefully cushioned from want and trouble of any sort; and through her marriage she finally gains these things. To him it means work done for humanity, knowledge increased, and suffering relieved to the full extext of his power; and he has to abandon the aim he had set for himself. The failure of Lydgate's life is not less complete and irretrievable because the world does not see it. Doubtless other people besides Rosamond thought he had done very well when he had established a reputation for the treatment of gout—"an ailment which," as the author remarks, "usually has wealth behind it." It is a pitiful story, all the more pitiful that it is such an every-day tragedy. We can all point to men we know who have done less for the world than their youth promised, simply because those dependent on them could not see beyond the material side of things; and desired for them not the power and opportunity to do good work, but merely those rewards which the highest labour does not inevitably command.

*(To be continued.)*

